# Development of a Molecular Adjuvant to Enhance Antigen-Specific CD8^+^ T Cell Responses

**DOI:** 10.1038/s41598-018-33375-1

**Published:** 2018-10-09

**Authors:** Benedict R. Halbroth, Sarah Sebastian, Hazel C. Poyntz, Migena Bregu, Matthew G. Cottingham, Adrian V. S. Hill, Alexandra J. Spencer

**Affiliations:** The Jenner Institute, University of Oxford, ORCRB, Roosevelt Drive, Oxford, United Kingdom

## Abstract

Despite promising progress in malaria vaccine development, an efficacious subunit vaccine against *P*. *falciparum* remains to be licensed and deployed. This study aimed to improve on the immunogenicity of the leading liver-stage vaccine candidate (ChAd63-MVA ME-TRAP), known to confer protection by eliciting high levels of antigen-specific CD8^+^ T cells. We previously showed fusion of ME-TRAP to the human MHC class II invariant chain (Ii) could enhance CD8^+^ T cell responses in non-human primates, but did not progress to clinical testing due to potential risk of auto-immunity by vaccination of humans with a self-antigen. Initial immunogenicity analyses of ME-TRAP fused to subdomains of the Ii showed that the Ii transmembrane domain alone can enhance CD8^+^ T cell responses. Subsequently, truncated Ii sequences with low homology to human Ii were developed and shown to enhance CD8^+^ T cell responses. By systematically mutating the TM domain sequence, multimerization of the Ii chain was shown to be important for immune enhancement. We subsequently identified several proteins from a variety of microbial pathogens with similar characteristics, that also enhance the CD8^+^ T cell response and could therefore be used in viral vector vaccines when potent cell mediated immunity is required.

## Introduction

Genetically engineered viral vectors are excellent inducers of strong CD8^+^ T cell responses, and recent progress in liver-stage malaria vaccine development with ChAd63-MVA vaccination regimens encoding ME-TRAP is very encouraging, with some degree of clinical efficacy in both malaria naïve and pre-exposed individuals^1,2^. Since protection against the liver-stage of malaria has been strongly associated with high numbers of antigen specific CD8^+^ T cells, it has been a priority to further increase the immunogenicity induced by vaccination with viral vectors. Numerous approaches, often using well-known adjuvants, have been explored, but failed during translation to clinical trials due to the lack of an effect in higher order species. However, several groups have now reported that genetic fusion of their vaccine antigen to the invariant chain (Ii) of MHC class II enhances antigen-specific CD4^+^ as well as CD8^+^ T cell responses across various animal species^3–12^. The exact mechanisms that leads to the observed adjuvanticity remain unclear, even though the interaction of Ii with HLA molecules is well-described.

Ii acts as (1) a scaffold for MHC class II assembly in the ER^13,14^, (2) a guardian to prevent endogenous peptides from binding to the MHC class II binding groove during early intracellular transport^15^, and (3) a leader to direct MHC class II to endolysosomal compartments directly via the Golgi apparatus or by recycling from the cell surface membrane^16^. An interaction between MHC class I, especially β2-microglobulin, and Ii as well as their co-localisation in endocytic compartments has been known since the 1990s^17–20^ and in 2002 Reber *et al*. demonstrated that transfection of HeLa cells with Ii leads to higher surface expression of MHC class I, suggesting a functional importance of the Ii-MHC-class-I-interaction^21,22^. This physiological significance in cross-presentation was then creatively demonstrated by Basha *et al*. in^21^. The Ii binds a fraction of newly synthesised MHC class I and directs them to endolysosomal compartments, thereby facilitating the binding of peptides which are derived from exogenous antigens^23^. Interestingly, the Ii therefore seems to interact with MHC II and MHC I molecules in a very similar way^24,25^.

Recent reports have now confirmed Ii adjuvanticity in non-human primates immunised with viral vectors encoding either an HCV or malaria antigen^3,11^. One of the last potential obstacles before clinical testing of the Ii as a molecular adjuvant are safety concerns, as the expression of Ii, a self-antigen, from a highly immunogenic viral vector could theoretically break immune-tolerance and induce an auto-immune response. These concerns were brought to attention when two publications documented autoantibodies targeting the CLIP region of the Ii^26,27^. Therefore in this study, two strategies were pursued to minimise the potential risk of inducing an auto-immune response by vaccination with the human Ii encoded in the viral vector. Firstly, the Ii molecule was truncated systematically to determine the minimal length of the Ii needed for its adjuvanticity. Secondly, the human Ii was replaced by various animal Ii sequences with low sequence homology to human. Ii variants were fused to ME-TRAP and expressed in viral vectors, the chimpanzee adenovirus 63 (ChAd63) and modified vaccinia Ankara (MVA), and tested for immunogenicity in animal models. Attempts to understand the mechanism of action causing adjuvanticity then led to the discovery of several novel adjuvants based on proteins of non-human origin.

## Results

### Ii Truncation to N-terminal 72 Amino Acids

**Figure 1:**
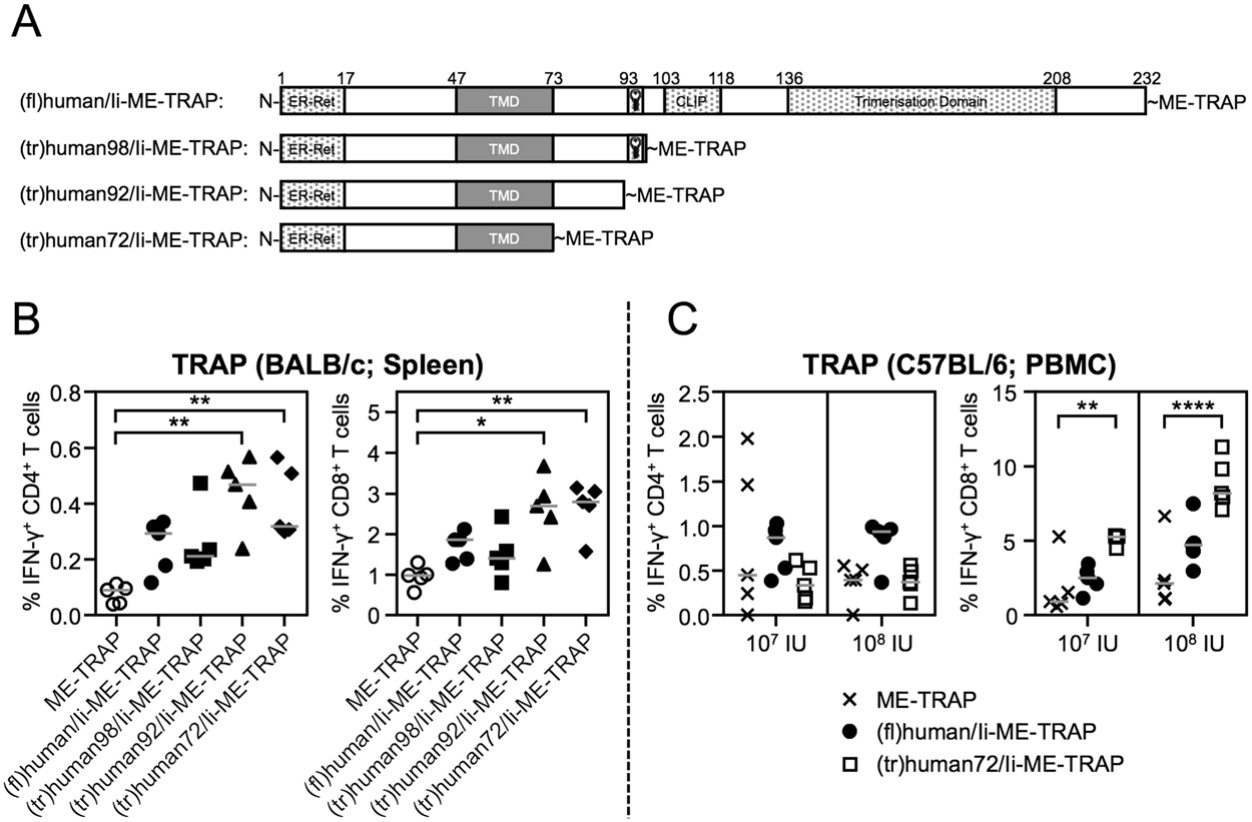
Initial truncation of Ii in Ii-ME-TRAP constructs. (**A**) The DNA sequence of the liver-stage antigen ME-TRAP was fused to the C-terminal end of either the full-length human p35 isoform of the invariant chain or Ii truncations of 98, 92, or 72 amino acid (aa) length. The full-length Ii encodes a transmembrane domain (TM, aa 47–72), the Ii-Key domain (aa 93–96), the CLIP domain (aa 103–117), as well as the C-terminal trimerisation domain (aa 136–207). (**B**) Immune response to ME-TRAP fused to Ii truncations. BALB/c mice were vaccinated IM with 10^7^ IU of ChAd63 vectors encoding unfused ME-TRAP or ME-TRAP fused to (fl)human/Ii-ME-TRAP, (tr)human98/Ii, (tr)human92/Ii, or (tr)human72/Ii. Spleens were harvested two weeks later and T cell responses to TRAP were measured by ICS. Points represent individual mice after subtraction of background responses and lines represent the median. (**C**) C57BL/6 mice were immunised IM with 10^7^ or 10^8^ IU of ChAd63 encoding unfused ME-TRAP, (fl)human/Ii-ME-TRAP or (tr)human72/Ii-ME-TRAP. Blood PBMC were analysed two weeks post vaccination with T cell responses measured by ICS. Data was analysed with a one-way analysis of variance with Dunn’s multiple comparison post-test. Asterisks denote the level of statistical significance (*p < 0.05; **p < 0.01; ****p < 0.0001).

The ME-TRAP antigen construct, which has been used in numerous clinical trials^1,2^, comprises a human codon-optimised multi-epitope string (ME) fused to the native *P*. *falciparum* T9/96 strain sequence of TRAP. When fused to Ii sequences, the nucleotides 1–75 of TRAP, which encodes a predicted signal peptide, were deleted in order to prevent hydrolysis of the Ii from TRAP (if signal peptide cleavage were to occur). This deletion within ME-TRAP itself did not have an impact on immunogenicity when expressed in ChAd63 vectors (Fig. S1). ME-TRAP was then fused to the C-terminal end of the full-length human p35 isoform of the invariant chain and named (fl)human/Ii-ME-TRAP. To identify the essential parts of Ii for its adjuvanticity, the Ii sequence was systematically truncated. In the first set of Ii-ME-TRAP constructs, the Ii was truncated to its N-terminal 98 aa, 92 aa, or 72 aa, respectively (Fig. 1A). The CLIP region of the Ii has repeatedly been shown to be crucial for MHC association, therefore it was important to establish its relevance as an adjuvant^28,29^.

To determine whether Ii truncations fused to ME-TRAP could enhance antigen-specific T cell responses, BALB/c mice were vaccinated with 10^7^ IU of ChAd63 vectors intramuscularly (IM), with spleens harvested at the peak of the response^30^ 14 days later, and splenocytes stimulated with a single pool of overlapping peptides covering the full TRAP sequence to measure production of cytokines by intracellular cytokine staining (ICS). Both the 72aa and 92aa truncated [Ii]-ME-TRAP vectors induced statistically significantly higher TRAP-specific immune response compared to unfused ME-TRAP (Fig. 1B). These high levels of TRAP-specific CD8^+^ T cell responses were induced even though the ME string of ME-TRAP contains a strong immunodominant H-2^d^ restricted epitope from CSP (Pb9) (Fig. S2), which has been shown to hamper the analysis of TRAP-specific immunogenicity in BALB/c mice^30^.

When the immunogenicity of [Ii]-ME-TRAP fusion were tested in C57BL/6 mice, TRAP-specific CD8^+^ T cell responses were significantly higher in mice immunised with ChAd63 vectors expressing the (tr)human72/Ii-ME-TRAP, regardless of the vaccination dose. MVA vaccines expressing (tr)human72/Ii-METRAP constructs did not show a significant enhancement of CD4^+^ or CD8^+^ T cell responses (Fig. S3), consistent with previous data^11^. Given that MVA vaccination of C57BL/6 mice induces higher CD4^+^ than CD8^+^ responses to TRAP, in contrast to ChAd63 vaccination, the lack of enhancement of TRAP-specific responses when fused to the invariant chain could be related to the MVA vector which preferentially induces CD4^+^ T cell responses.

### Ii adjuvanticity in ChAd63-MVA prime-boost regimens

**Figure 2:**
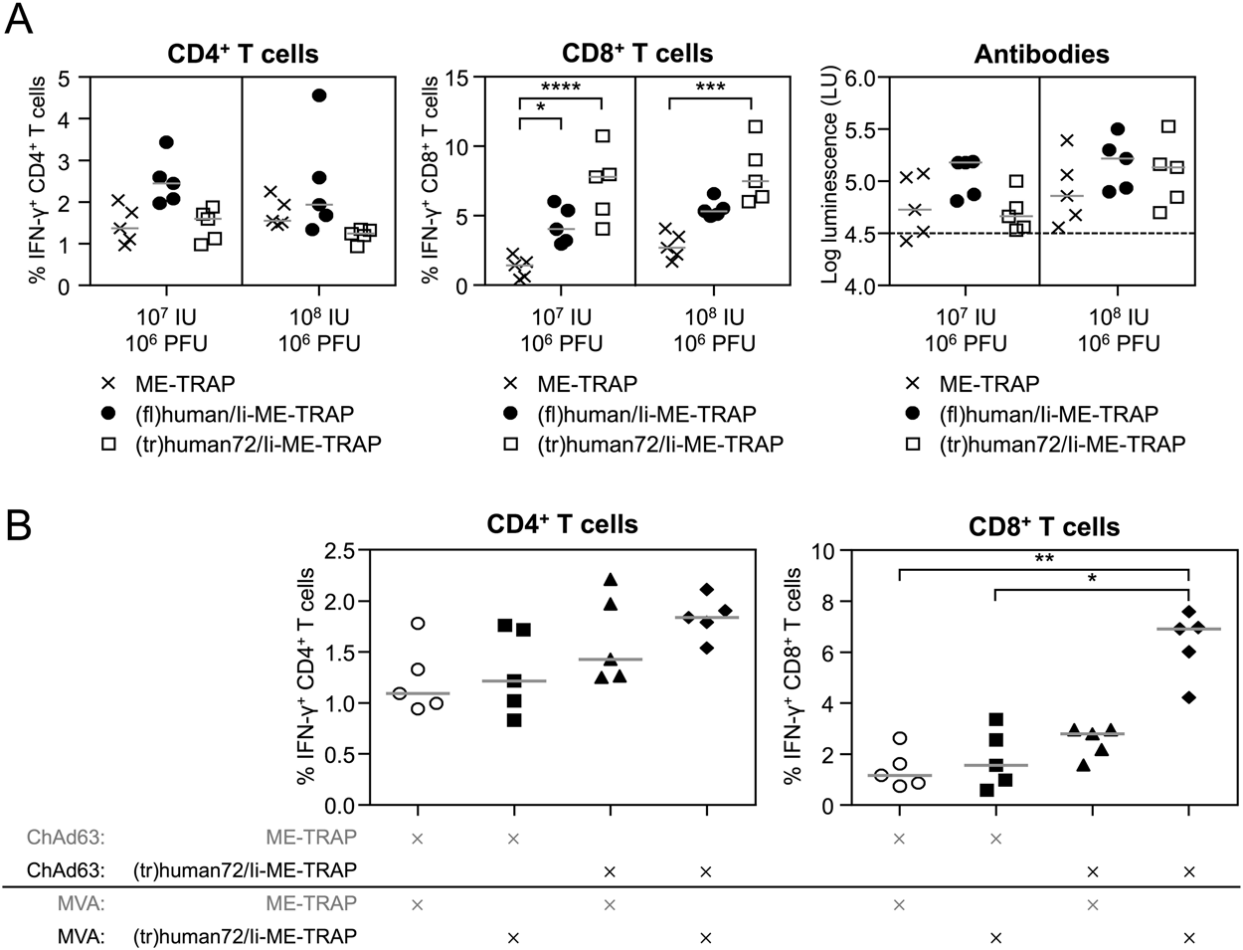
Truncated Ii in ChAd63-MVA prime-boost regimens. (**A**) Five C57BL/6 mice per groups were immunised IM with 10^8^ IU or 10^7^ IU of ChAd63 encoding unfused ME-TRAP, (tr)human72/Ii-ME-TRAP, or (fl)human/Ii-ME-TRAP and seven weeks later boosted with either 10^6^ PFU MVA encoding the identical insert. Two weeks post boost, a blood sample was taken for antibody analyses, and spleens harvested and analysed for TRAP-specific T cell responses by ICS. (**B**) C57BL/6 mice were immunised IM with 10^6^ IU of ChAd63 encoding unfused ME-TRAP or (tr)human72/Ii-ME-TRAP and eight weeks later boosted with either 10^6^ PFU MVA-ME-TRAP or MVA-(tr)human72/Ii-ME-TRAP as indicated below the x-axes. Spleens were harvested one week later and T cell responses to TRAP were measured by ICS. Points represent individual mice after subtraction of background responses and lines represent the median. Data was analysed with a one-way analysis of variance with Dunn’s multiple comparison post-test. Asterisks denote the level of statistical significance (*p < 0.05; **p < 0.01; ***p < 0.001; ****p < 0.0001).

To investigate whether the improved adjuvanticity mediated by the truncated Ii version could enhance immunogenicity in a prime-boost regimen, C57BL/6 mice were vaccinated with 10^7^ or 10^8^ IU of ChAd63 encoding unfused ME-TRAP, (fl)human/Ii-ME-TRAP or (tr)human72/Ii-ME-TRAP and boosted seven weeks later with 10^6^ PFU MVA encoding the identical inserts (Fig. 2A). Ii truncation TRAP-specific CD8^+^ T cell responses were significantly increased, compared to non-adjuvanted ME-TRAP, when mice were immunised with vectors encoding (tr)human72/Ii-ME-TRAP. In contrast, Ii truncation did not enhance CD4^+^ immunogenicity or antibody response to TRAP.

We further analysed whether the inclusion of (tr)human/Ii in both viral vectors (ChAd63 and MVA) was beneficial compared to vaccination regimens in which none or only one of both vectors (ChAd63 or MVA) was adjuvanted by (tr)human/Ii (Fig. 2B). CD8^+^ T cell responses were significantly increased, compared to the non-adjuvanted control, when ME-TRAP was fused to the (tr)human/Ii adjuvant in both viral vectors, no difference in CD4^+^ T cell responses were observed. Interestingly, when the adjuvant was not present in the ChAd63 prime, ME-TRAP fused to Ii did not significantly improve CD8^+^ T cell responses (Fig. 2B).

To assess the ability of truncated Ii to augment TRAP-specific immune responses in non-human primates (NHP), two groups of male rhesus macaques were immunised with ChAd63 and MVA encoding unfused ME-TRAP or adjuvanted (tr)human72/Ii-ME-TRAP. TRAP-specific ELISpot responses demonstrated a peak in immunogenicity one week post MVA boost and with responses still detectable in almost all animals at week 16. However, significant differences between the two groups were not observed at any timepoint (Fig. S4A). TRAP specific responses measured by IFN-γ ICS at the peak timepoint after ChAd63 prime (week 2) or MVA boost (week 9) demonstrated very low TRAP-specific CD4^+^ and CD8^+^ T cell responses in almost all animals (11/12 macaques). One week post MVA boost (week 9), the TRAP-specific CD4^+^ T cell response was increased compared to week 2, with no significant difference between the two vaccine groups. In contrast, the frequency of antigen-specific CD8^+^ T cell responses did not increase after MVA boost in either group. This was unexpected as MVA expressing unfused ME-TRAP had been shown to boost TRAP-specific CD8^+^ T cell results in two previous macaque studies^11,31^. The lack of CD8^+^ T cell boost by MVA vaccination (despite a boost of CD4^+^ T cells) suggests that the ChAd63 vaccination failed to prime rhesus macaques and therefore the post-MVA responses are more representative of a primary response to MVA. If so, the lack of adjuvant effect of Ii observed in rhesus macaques is consistent with the results in mice where ME-TRAP fused to Ii fails to enhance T cell responses after vaccination with MVA, or when the (tr)human72/Ii-ME-TRAP is only expressed in the MVA vector (Fig. 2B), due to the vectors preponderance to induce CD4^+^ T cells.

### Further Ii Truncations

**Figure 3:**
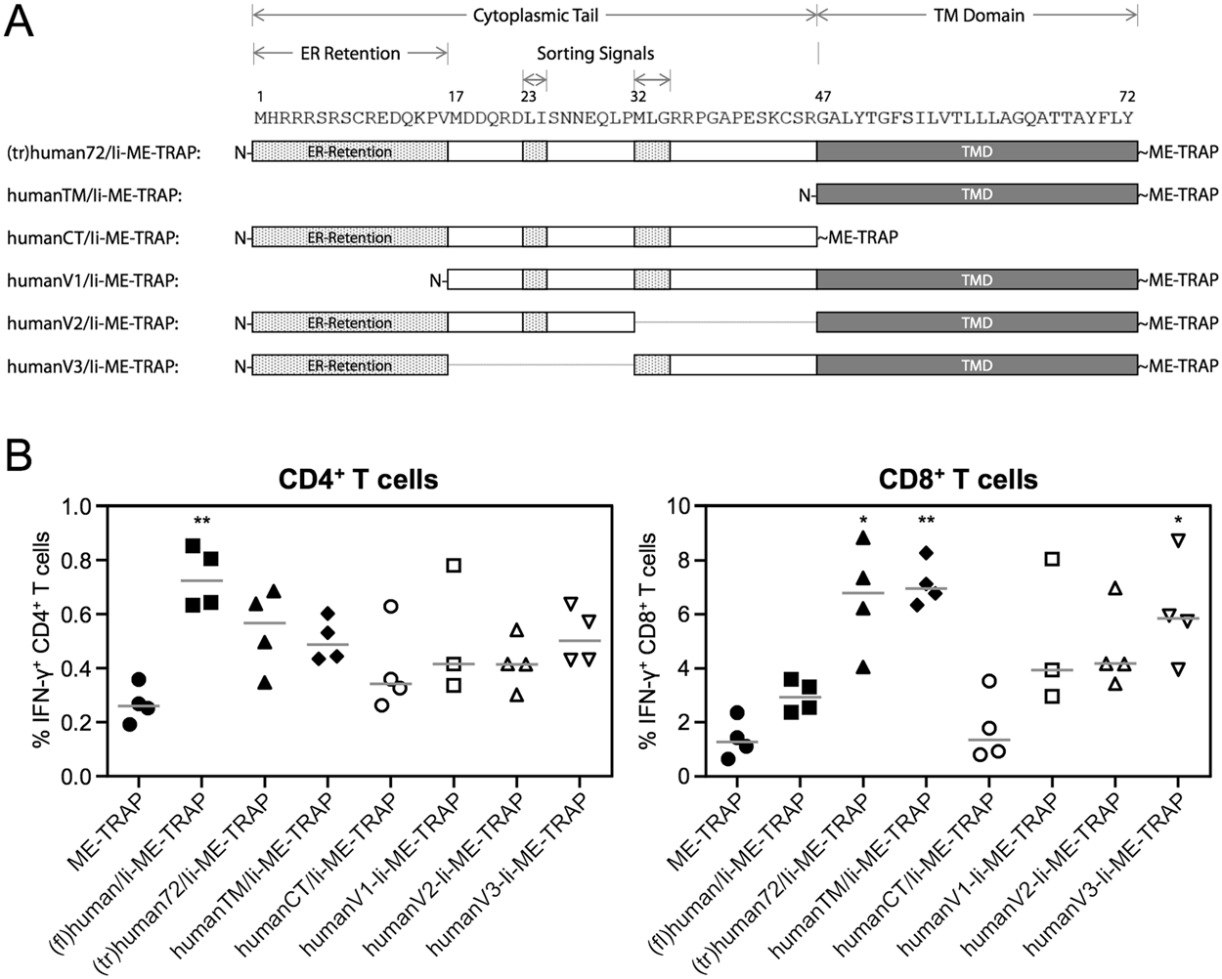
Ii TMD is essential for adjuvanticity. (**A**) Further truncations of the Ii. The DNA sequence of the liver-stage antigen ME-TRAP was fused to truncated human invariant chain sequences. As prior results indicated that strong CD8^+^ T cell responses could be induced with a 72 amino acid long sequence of the Ii chain, new constructs with further truncations were designed as indicated. (**B**) C57BL/6 mice were immunised with 10^7^ IU ChAd63 vectors and spleens were harvested two weeks later. T cell responses to a single pool of TRAP peptides were analysed by ICS. The percentage of CD4^+^ and CD8^+^ T cells positive for IFN-γ are shown. Single points indicate T cell responses of individual mice and lines denote the median response per group. Data was analysed with a one-way analysis of variance with Dunn’s multiple comparison post-test. Asterisks denote the level of statistical significance when compared to the control ME-TRAP vaccinated group (*p < 0.05; **p < 0.01).

Based on the encouraging results obtained in mice, further Ii constructs were designed to identify the minimal sequence of the Ii chain required to enhance CD8^+^ T cell responses. The different constructs encoded either only the Ii transmembrane domain (humanTM/Ii-ME-TRAP), the cytoplasmic tail (humanCT/Ii-ME-TRAP) or had deletions of either the ER retention site (humanV1/Ii-ME-TRAP) or one of the sorting signals (humanV2/Ii-ME-TRAP and humanV3/Ii-ME-TRAP) (Fig. 3A).

Two weeks after immunisation the highest CD8^+^ T cell responses were observed when ME-TRAP was fused to (tr)human72/Ii or humanTM/Ii (Fig. 3B). Both induced an approximately 5.5-fold and statistically significant increase in the frequency of IFN-γ producing CD8^+^ T cells compared to unfused ME-TRAP. In the absence of the transmembrane domain (humanCT/Ii-ME-TRAP), no difference to the unfused ME-TRAP construct was observed, demonstrating the importance of the transmembrane domain for the adjuvant effect. The remaining three constructs, humanV1-Ii-ME-TRAP, humanV2-Ii-ME-TRAP, and humanV3-Ii-ME-TRAP induced responses equivalent to the response from (fl)human/Ii-ME-TRAP, but a lower number than humanTM/Ii-ME-TRAP. In contrast, mice vaccinated with (fl)human/Ii-ME-TRAP expressing ChAd63 was the only group of mice to show a significant increase in CD4^+^ T cells responses compared to the control.

### Why does Ii Truncation Enhance the Immune Response?

When (fl)human/Ii was truncated to the 72 aa long (tr)human72/Ii and fused to ME-TRAP, the induced antigen-specific CD8^+^ T cell response was significantly increased compared to the full-length Ii construct, (fl)human/Ii-ME-TRAP. Given the increased size of (fl)human/Ii-ME-TRAP relative to ME-TRAP, one possible explanation is that a competing CD8^+^ T cell response against the xenogenic (fl)human/Ii, could be reducing the response to the ME-TRAP protein. To test this hypothesis two different strategies were pursued.

**Figure 4:**
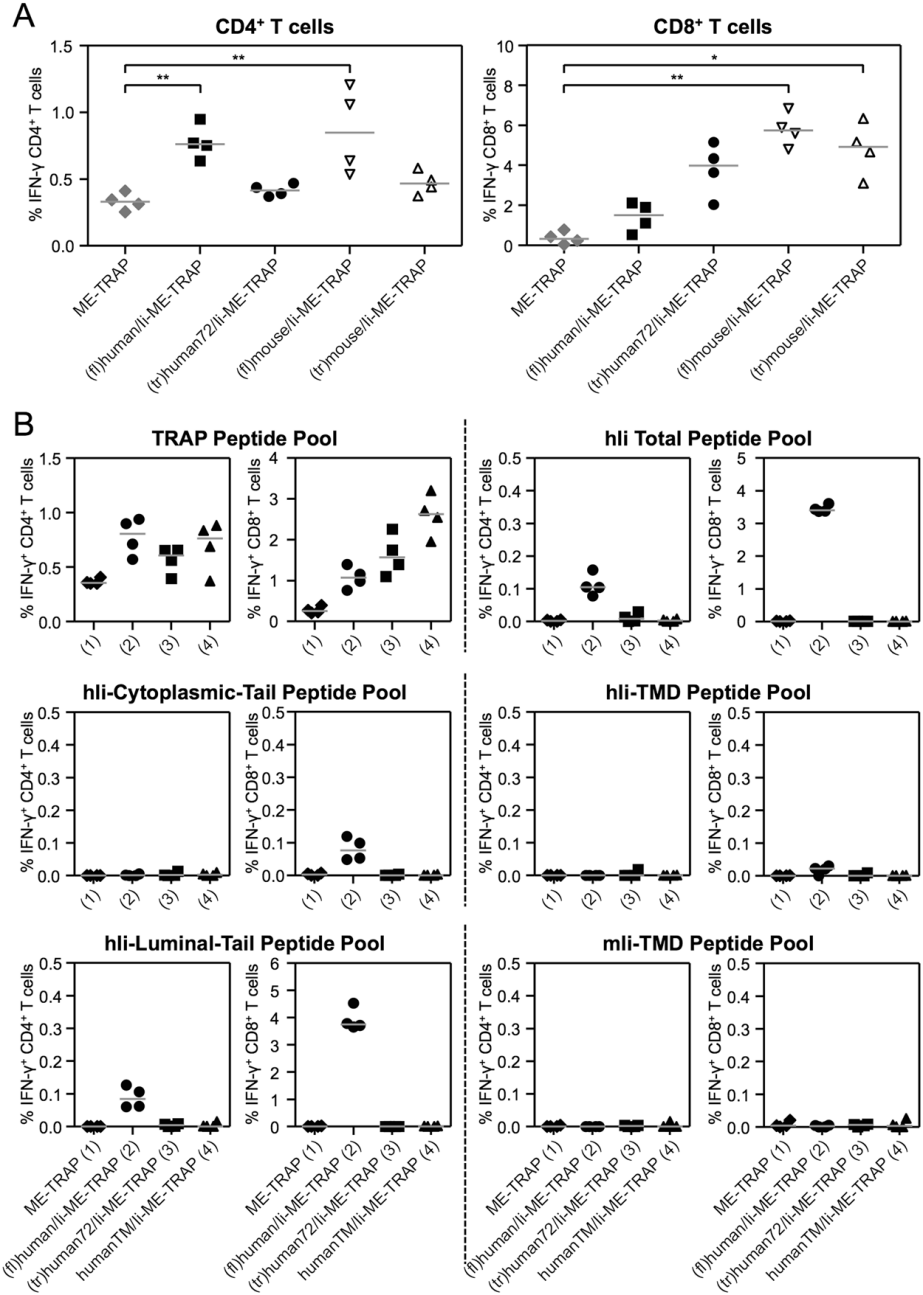
Competing immunogenicity. (**A**) C57BL/6 mice were immunised with 10^7^ IU ChAd63 vectors and spleens were harvested two weeks later. T cell responses to a TRAP peptide pool were analysed by ICS. The percentage of CD4^+^ and CD8^+^ T cells positive for IFN-γ are shown. Data was analysed with a one-way analysis of variance with Dunn’s multiple comparison post-test. Asterisks denote the level of statistical significance between indicated groups (*p < 0.05; **p < 0.01). (**B**) C57BL/6 mice were immunised with 10^8^ IU ChAd63 vectors and spleens were harvested two weeks later. T cell responses against peptide pools covering TRAP (TRAP Peptide Pool), the full human Ii sequence (hIi Total Peptide Pool), only its cytoplasmic tail (hIi Cytoplasmic-Tail Peptide Pool), only its TMD (hIi-TMD Peptide Pool), only its luminal domain (hIi-Luminal-Tail Peptide Pool), or the mouse Ii TMD sequence (mIi-TMD Peptide Pool) were analysed by ICS. The percentage of CD4^+^ and CD8^+^ T cells positive for IFN-γ are shown. Single points indicate T cell responses of individual mice and lines denote the median response per group.

Firstly, full-length and truncated versions of mouse Ii sequences were fused to ME-TRAP and ChAd63 viral vectors produced and tested in C57BL/6 mice. Both vectors expressing ME-TRAP fused to full-length Ii sequences, (fl)human/Ii-ME-TRAP or (fl)mouse/Ii-ME-TRAP, induced similarly strong TRAP-specific CD4^+^ T cell responses, which were significantly higher compared to the response induced by unfused ME-TRAP (Fig. 4A), while truncated mouse or human Ii chain did not enhance CD4^+^ T cell responses. Consistent with previous observations, a stronger antigen-specific CD8^+^ T cell response was induced by (tr)human72/Ii-ME-TRAP compared to (fl)human/Ii-ME-TRAP. In contrast, no difference in the frequency of antigen specific responses between the full-length or truncated mouse Ii responses were observed and both constructs induced significantly increased CD8^+^ T cell responses compared to the unadjuvanted ME-TRAP control group (Fig. 4A).

Therefore, in a second experiment, we tested whether vaccination with Ii could induce an immune response against the Ii sequence (Fig. 4B). C57BL/6 mice vaccinated with ChAd63 vectors expressing unfused ME-TRAP or ME-TRAP fused to either full-length human Ii [(fl)human/Ii], the truncated 72 aa long version of human Ii [(tr)human72/Ii] or the transmembrane domain of human Ii [humanTM/Ii] were analysed for the response against a human Ii chain peptides. Interestingly, an immune response directed against the human Ii was only observed when mice were immunised with ME-TRAP fused to the full-length human Ii sequence, with Ii-specific CD4^+^ and CD8^+^ T cell responses directed primarily against epitopes within the luminal domain of the Ii. A low CD8^+^ T cell response against the Ii cytoplasmic tail and TMD was also detected when mice were immunised with (fl)human/Ii-ME-TRAP.

Taken together the data suggest that immune responses to the Ii can impact on its ability to enhance the antigen-specific CD8^+^ T cell response. Therefore truncated versions of non-homologous Ii sequences would not only reduce theoretical safety concerns of vaccination with a self-antigen, but may show greater adjuvant capacity than their full-length equivalents.

### Xenogenisation of Ii

The second strategy to reduce the potential risk of breaking immuno-tolerance by vaccination of humans with a human Ii sequence, was to identify and design Ii aa sequences with low homology to the human Ii. The NCBI protein database was screened for Ii sequences from a large variety of animal species and sub-groups, such as birds, fish, mammals, or amphibians. Sequences were compared to the full-length human sequences to determine their homology using the ClustalW algorithm in DNAStar® Megalign (Table S1). The two Ii sequences with the lowest homology to (fl)human/Ii, namely (fl)trout/Ii and (fl)shark/Ii, were fused to the N-terminal end of ME-TRAP and produced in ChAd63 viral vectors. As our initial immunogenicity data suggested that the luminal domain of the Ii is not needed for adjuvanticity, Ii sequences from various animal species were truncated (tr) according to the equivalent length of (tr)human72/Ii and analysed for homology to (tr)human72/Ii or (tr)mouse/Ii using the ClustalW algorithm in DNAStar® Megalign (Table S2). Constructs with high sequence homology with (tr)human72/Ii were not further considered. Highlighted sequences were produced in ChAd63 vectors to obtain *in vivo* immunogenicity data.

**Figure 5:**
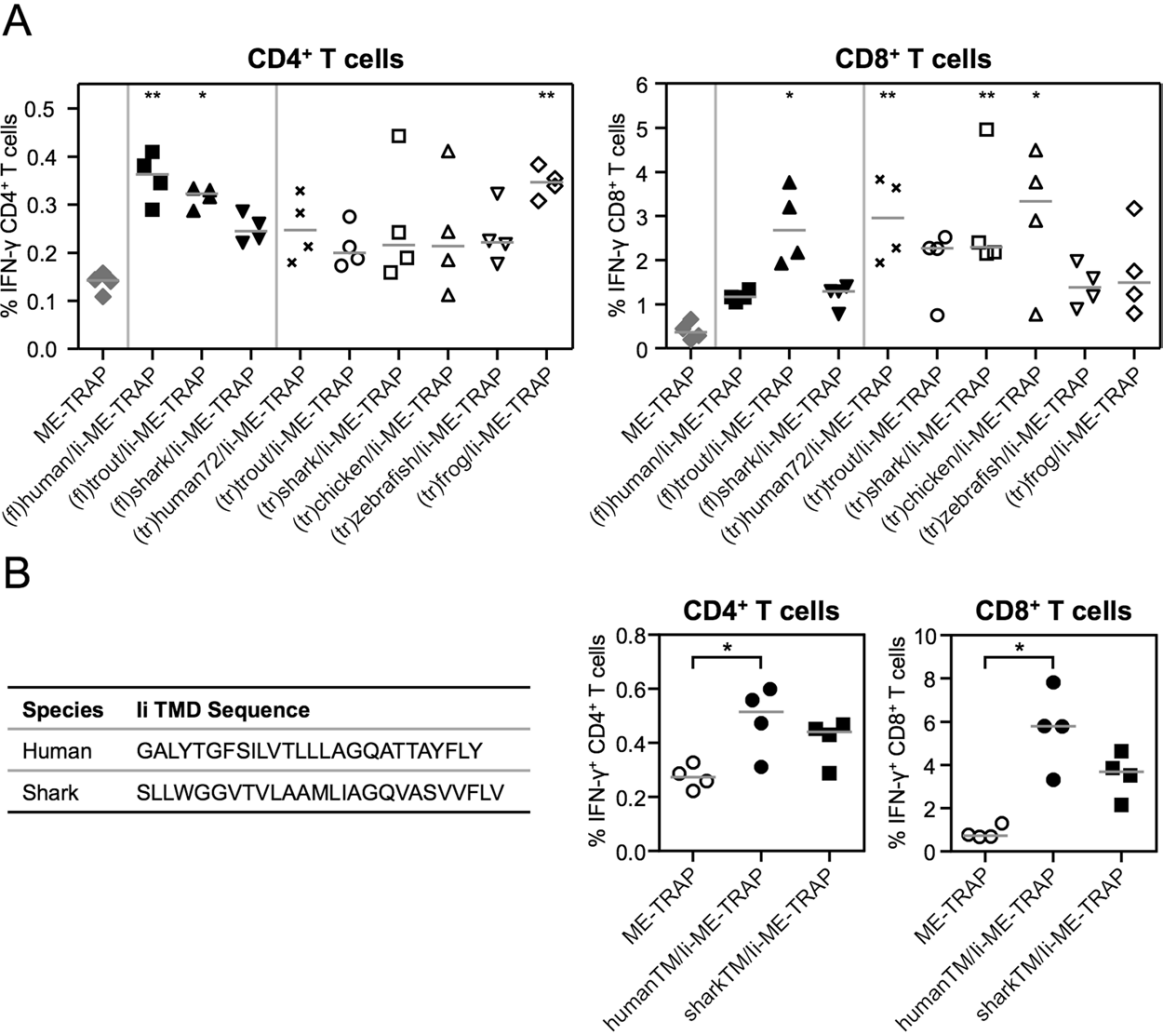
Adjuvanticity of xenogenised Ii. C57BL/6 mice were immunised with 10^7^ IU ChAd63 vectors encoding ME-TRAP adjuvanted by (**A**) full-length or truncated sequences of xenogenised Ii, (**B**) the TMD of human or shark Ii, or unfused ME-TRAP as control. Spleens were harvested two weeks later and T cell responses to a TRAP peptide pool analysed by ICS. The percentage of CD4^+^ and CD8^+^ T cells positive for IFN-γ are shown. Single points indicate T cell responses of individual mice and lines denote the median response per group. Data was analysed with a one-way analysis of variance with Dunn’s multiple comparison post-test. Asterisks denote the level of statistical significance when compared to the control ME-TRAP vaccinated group (*p < 0.05; **p < 0.01).

C57BL/6 mice were vaccinated with ChAd63 vectors encoding full-length or truncated xenogenised Ii-ME-TRAP fusion constructs and splenocytes analysed by ICS two weeks later. When CD8^+^ T cell responses were compared to the control group, significantly higher frequencies of CD8^+^IFN-γ^+^ T cells were observed when ME-TRAP was fused to (fl)trout/Ii-ME-TRAP (p < 0.05), (tr)human72/Ii (p < 0.01), (tr)shark/Ii (p < 0.01), or (tr)chicken/Ii (p < 0.05), respectively (Fig. 5A). A significant increase in TRAP-specific CD4^+^IFN-γ^+^ T cell responses were observed when ME-TRAP was fused to (fl)human/Ii (p < 0.01), (fl)trout/Ii (p < 0.05), or (tr)frog/Ii (p < 0.01), respectively.

As the transmembrane domain (TMD) of the human Ii showed a similarly strong ability to enhance the response compared to (tr)human72/Ii (Fig. 3), Ii TMD of numerous animal species were compared. The analysis revealed that the Ii TMD sequence is highly conserved between animal species. This becomes more obvious when amino acids with similar biochemical characteristics are colour coded in groups as in Figure S5. Still, when compared to the humanTM/Ii using MUSCLE or ClustalW algorithms, sharkTM/Ii has a very low sequence homology of 35% (Fig. 5B). Importantly, the shark Ii TMD (sharkTM/Ii) has only three identical consecutive amino acids in common with the humanTM/Ii. For this reason, sharkTM/Ii was also fused N-terminally to ME-TRAP to investigate its ability to enhance the immune response. TRAP-specific CD4^+^ and CD8^+^ T cell responses were found to be increased when ME-TRAP was fused to sharkTM/Ii compared to non-adjuvanted ME-TRAP (Fig. 5B), but immunogenicity was lower compared to humanTM/Ii-ME-TRAP.

### Understanding the mechanism of Ii enhancement

Previous *in vitro* results^5,8^ suggested that the increased CD8^+^ T cell response following antigen fusion to Ii was due to a direct effect on transduced DC leading to a more efficient presentation of antigen derived peptides on MHC class I (and II). To understand if antigen fusion to Ii had an impact on intracellular trafficking of the antigen, immunohistological analysis of transfected A549 cells (human adenocarcinoma epithelial cell line) was performed with antigen distribution within the cells detected by immunofluorescent staining. Unfused ME-TRAP was primarily located on the cell surface (Fig. S6). When the predicted signal peptide of TRAP was deleted (w/o_eSP), TRAP was distributed diffusely across the cell. Fusion of ME-TRAP (which also lacks the TRAP signal peptide) to the full-length human Ii led to a significant alteration in TRAP expression, with the most intense fluorescence detected around the cell nucleus. This distinct localisation pattern was also observed when ME-TRAP was fused to other Ii truncations. A notable exception was humanCT/Ii-ME-TRAP, which did not enhance CD8^+^ T cell responses. Even though the most intense fluorescence could still be detected intracellularly, staining of the plasma membrane was also more pronounced. A549 cells were also transfected with plasmid DNA encoding truncated Ii sequences from various animal species (xenogenised Ii). In general, TRAP localisation was similar to (tr)human72/Ii-ME-TRAP. When cells were transfected with (tr)chicken/Ii-ME-TRAP or (tr)trout/Ii-ME-TRAP expressing constructs, TRAP expression was found to accumulate in small round-shaped conglomerations. Although this pattern of expression was not unique to these two constructs, it occurred at a higher frequency.

One could also argue that *in vivo* T cell immunogenicity enhancement by Ii is not caused by a direct effect of antigen processing in DC, as the majority of transduced cells after an intramuscular injection are not DC, but muscle cells or fibroblasts inside the muscle tissue. For T cell priming, antigen stability could then be of higher importance in order to allow effective cross-presentation^32,33^. Ii trimerisation could be crucial in this regard, as multimers tend to be more stable to proteolysis. To investigate the importance of Ii trimerisation, a mutated version of (tr)human72/Ii-ME-TRAP was cloned, in which a glutamine was replaced by an alanine in position 64 by site directed mutagenesis and named (tr)humanQ64A/Ii-ME-TRAP, a mutation previously shown to disrupt Ii trimerisation^34^. However this mutation did not affect the ability of the Ii chain to enhance responses, as both CD4^+^ and CD8^+^ T cell responses were of a similar magnitude to unmutated (tr)human72/Ii-ME-TRAP (Fig. S7A). To confirm whether the introduced mutation had an effect on antigen multimerisation, western blot analysis was performed on HEK293 cells transfected with vectors encoding unfused or adjuvanted ME-TRAP constructs. All lysate samples from cells transfected with ME-TRAP variants showed at least two bands on the western blot, one of expected length and one slightly smaller (possibly due to partial post-translational modifications or expression from alternative translation initiation sites). Under non-reducing conditions, ME-TRAP appeared to multimerise/aggregate when fused to (fl)human/Ii, (tr)human72/Ii, humanTM/Ii, and also (tr)humanQ64A/Ii (Fig. S7B). Despite the reducing conditions, ME-TRAP still formed dimers when fused to these four Ii variants, suggesting an extraordinary multimer stability (Fig. S7C). Importantly, dimerisation was also seen when the Ii-TMD was mutated ((tr)humanQ64A/Ii-ME-TRAP). Multimerisation was therefore not disrupted by this mutation as had previously been reported.

**Figure 6:**
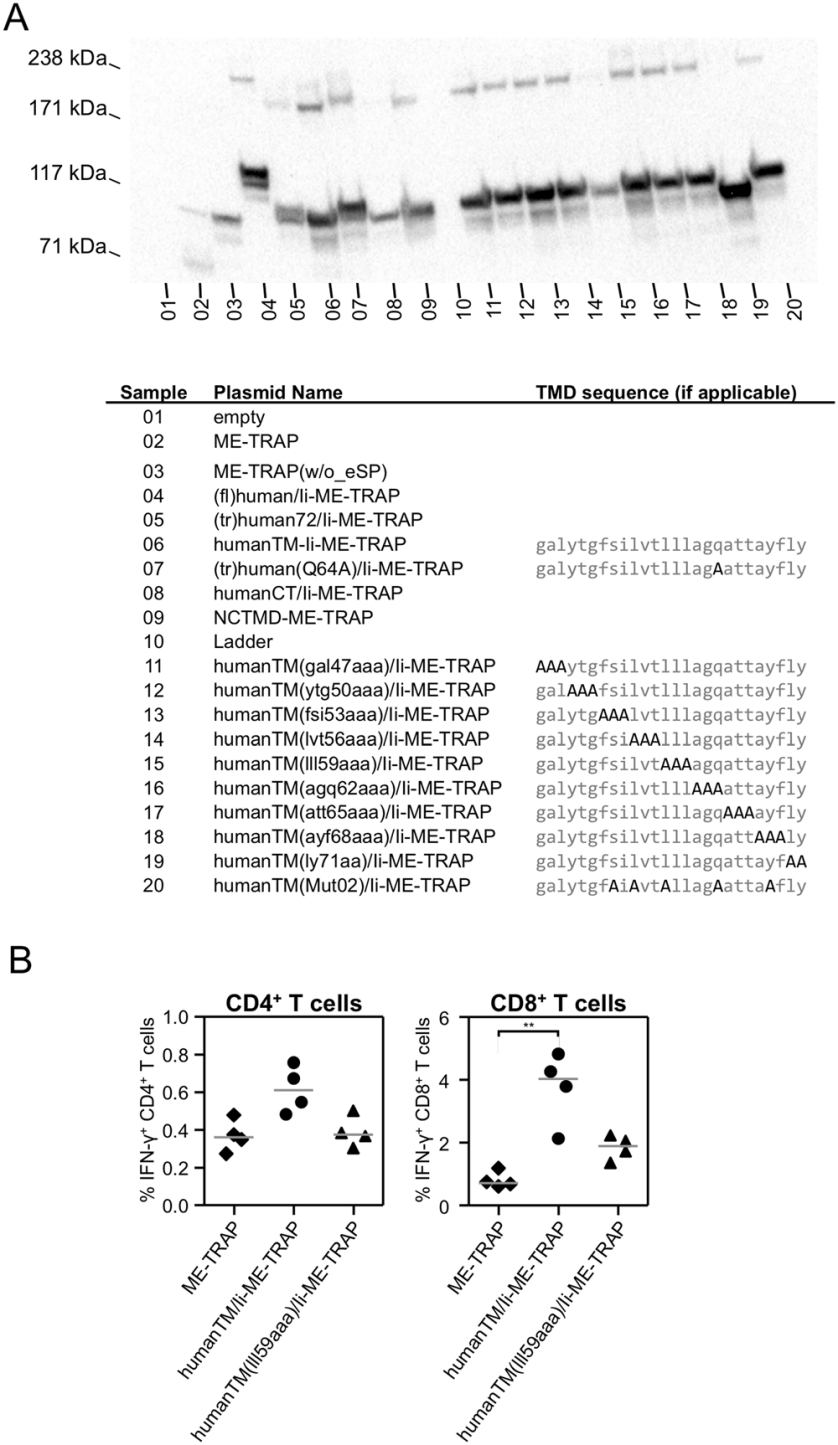
Western blot and immunogenicity of mutated Ii TMD. (**A**) HEK293 cells were transfected with plasmid DNA as shown in the table, which shows the sample name and sequence of Ii TMD if applicable (mutated amino acids are highlighted in bold and black). Cell lysates were analysed in reducing conditions (LDS loading buffer + 10% 2-Mercaptoethanol, 85 °C, 5 min) using a polyacrylamide gel and western blot stained with polyclonal serum against ME-TRAP. (**B**) C57BL/6 mice were immunised with 10^8^ IU ChAd63 vectors and spleens were harvested two weeks later. T cell responses to a single TRAP peptide pool were analysed by ICS. The percentage of CD4^+^ and CD8^+^ T cells positive for IFN-γ are shown. Single points indicate T cell responses of individual mice and lines denote the median response per group. Data was analysed with a one-way analysis of variance with Dunn’s multiple comparison post-test. Asterisks denote the level of statistical significance when compared to the control group vaccinated with ME-TRAP (**p < 0.01).

To determine if mutations at other locations across the TMD could stop Ii multimerisation and thus the adjuvant capacity of the Ii chain TM domain, the TMD of humanTM/Ii-ME-TRAP in the pENTR4-LPTOS plasmid was systematically mutated (Fig. 6A) and analysed by a western blot. One of the mutated candidates, humanTM(ly71aa)/Ii-ME-TRAP, had a N-terminal deletion (as confirmed by Sanger sequencing), which resulted in translation of a smaller protein. Another construct (humanTM(lll59aaa)/Ii-ME-TRAP) showed a lower tendency to multimerise. Therefore, a ChAd63 viral vector encoding the mutated humanTM(lll59aaa)/Ii-ME-TRAP was produced to assess its immunogenicity in C57BL/6 mice (Fig. 6B). Antigen-specific CD8^+^ T cell responses were not significantly increased when ME-TRAP was fused to mutated humanTM(lll59aaa)/Ii, suggesting multimerisation of the TMD of Ii chain is a critical for Ii to enhance T cell responses.

### Novel TMD as Molecular Adjuvants

**Figure 7:**
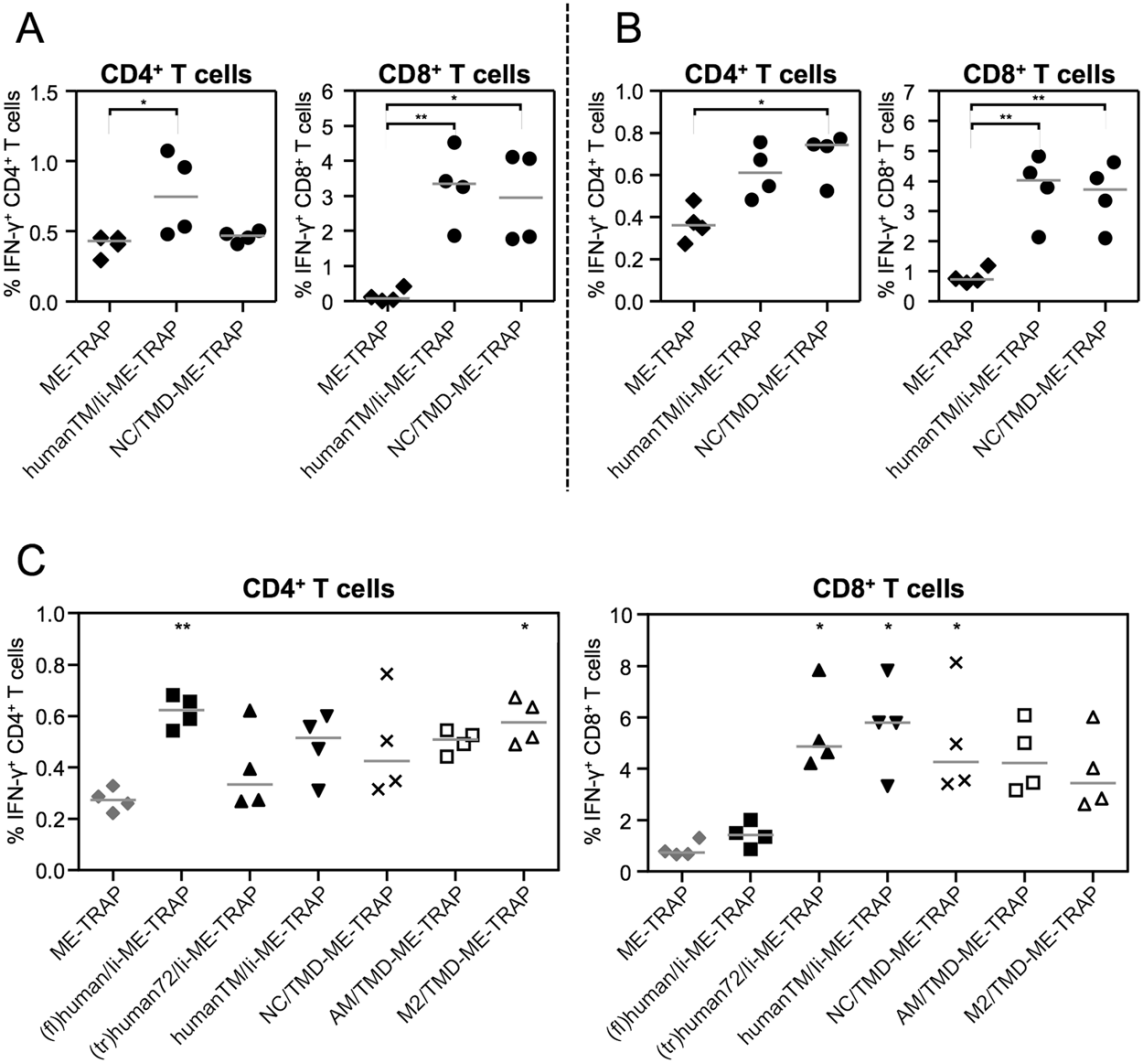
Immunogenicity of novel molecular adjuvants. C57BL/6 mice were immunised with ChAd63 vectors (10^7^ IU in **A** and **C**; 10^8^ IU in **B**) and spleens were harvested two weeks later. T cell responses to a single TRAP peptide pool were analysed by ICS. The percentage of CD4^+^ and CD8^+^ T cells positive for IFN-γ are shown. Single points indicate T cell responses of individual mice and lines denote the median response per group. Data was analysed with a one-way analysis of variance with Dunn’s multiple comparison post-test. Asterisks denote the level of statistical significance when compared to the control group vaccinated with ME-TRAP (*p < 0.05; **p < 0.01).

In the hope of identifying other potential molecular adjuvants, the literature was screened for TMD of non-human proteins which are known to trimerise or multimerise. Excitingly, the TMD of paramyxoviridae fusion proteins have recently been described to self-associate into trimeric complexes in the absence of the rest of the protein^35^, thus the 21 aa long TMD sequence of the fusion protein of one paramyxovirus (Newcastle disease virus) was fused to the N-terminal end of ME-TRAP and produced as a ChAd63 vector to compare its immunogenicity with existing unfused and adjuvanted ME-TRAP expressing vectors. At a dose of 10^7^ IU ChAd63 vectors, TRAP-specific CD8^+^ T cell responses induced by NC/TMD-ME-TRAP were of similar magnitude compared to that induced by humanTM/Ii-ME-TRAP (Fig. 7A) and significantly increase compared to unadjuvanted ME-TRAP. At a dose of 10^8^ IU ChAd63 vectors, both antigen-specific CD4^+^ and CD8^+^ T cell responses were statistically significantly increased compared to the response in mice vaccinated with the non-adjuvanted ME-TRAP (Fig. 7B).

Based on these results, two more TMD of non-human proteins were fused to ME-TRAP. One was the TMD sequence of another paramyxovirus (avian metapneumovirus, AM/TMD); the other was the TMD sequence of the M2 protein of influenza A, which is known to form tetramers in the absence of the rest of the M2 protein. TRAP-specific CD8^+^ T cell responses measured following vaccination with 10^7^ IU of ChAd63 encoding adjuvanted and unfused ME-TRAP showed that both, AM/TMD and M2/TMD, strongly enhanced CD8^+^ T cell responses, but not to the same level as humanTM/Ii (Fig. 7C). Only (fl)human/Ii-ME-TRAP and M2/TMD-ME-TRAP were shown to induce significantly higher TRAP-specific CD4^+^ T cell responses compared to unfused ME-TRAP.

## Discussion

The clinically most advanced viral vectored liver-stage malaria vaccine is based on a heterologous prime-boost vaccination regimen with ChAd63 and MVA encoding ME-TRAP. In contrast to most licensed vaccines, protection is correlated with the magnitude of antigen-specific CD8^+^ T cells^1^. However, the level of sterile protection observed in malaria-naïve vaccinees is only about 25%. Hence, for further improvement of vaccine efficacy, higher levels of cytotoxic T cells may be required. As the carrier viruses themselves have an adjuvantation effect^36^, it is difficult to further increase antigen-specific T cell responses by adding classic adjuvants^37^. We have previously assessed many potential molecular adjuvants in pre-clinical studies, such as TLR signalling molecules (TRAF, TRAM, TAK1)^38^, classical co-stimulatory molecules (4-1BBL, CD80, Raet1e)^39^, or cytokines (IL-15, IL-7, GM-CSF) (Spencer *et al*., unpublished data), but none sufficiently increased vaccine immunogenicity to a level that would merit progression to clinical testing.

In this study, the HLA class II associated invariant chain (Ii) was analysed and modified to improve immunogenicity and efficacy of viral vectored vaccines encoding ME-TRAP. Several groups have demonstrated that an extension of the C-terminal end of Ii with an antigenic sequence can facilitate enhanced activation of naïve CD4^+^ T cells^4–6^ as well as CD8^+^ T cell responses^5,6,8,9,40,41^. Prime-boost vaccination regimens with ChAd63 and MVA encoding ME-TRAP fused to the C-terminal end of the full-length human Ii have been reported to enhance CD4^+^ and CD8^+^ T cell responses in mice and NHP^14^. However, concerns that expressing the human Ii in viral vectors would break self-tolerance has slowed progression to clinical trials. These ideas were reinforced with recent data suggesting that autoantibodies against the CLIP domain of the Ii are associated with axial spondylitis^26,27^. To address these concerns, two strategies were pursued in this study: Ii truncation and Ii xenogenisation.

Initially, the Ii was truncated in a stepwise manner from its C-terminal end to a 72 aa long “truncated” Ii version and fused to the N-terminal end of ME-TRAP and immunogenicity compared. Strikingly, truncation of the Ii did not hinder its ability to enhance TRAP specific CD8^+^ T cell responses in BALB/c and C57BL/6 mice (Fig. 1), suggesting that the essential region of Ii is located in its N-terminal 72 aa. Using the most immunogenic clinical regimen, ChAd63 followed eight weeks later by an MVA boost, we also observed a significantly higher TRAP-specific immune response when both viral vectors encoded the truncated Ii [(tr)human72/Ii] fused to ME-TRAP compared to unfused ME-TRAP (Fig. 2). Although the truncated Ii failed to enhance the immune response in rhesus macaques, the absence of a boost in CD8^+^ T cell response after MVA vaccination, suggested that the ChAd63 vectors here surprisingly failed to adequately prime the animals, so a repeat experiment is required.

In an effort to identify the minimal sequence of Ii required to enhance T cell responses, the Ii sequence was further truncated and *in vivo* immunogenicity assessed. These experiments revealed that only the TMD of Ii, a peptide sequence of only 26 aa length, is needed to enhance T cell responses. (tr)human72/Ii-ME-TRAP as well as humanTM/Ii-ME-TRAP induced TRAP-specific CD8^+^ T cell responses. In contrast, when the TMD of the Ii was not expressed in the fusion construct (humanCT/Ii-ME-TRAP), T cell responses were comparable to the non-adjuvanted ME-TRAP vaccine. Further analysis revealed that T cell responses against the C-terminal end of the human Ii sequence were induced in mice. Therefore, an Ii-specific response competing with TRAP-specific responses may explain why the antigen-specific CD8^+^ T cell responses induced with the truncated Ii versions (namely (tr)human72/Ii-ME-TRAP and humanTM/Ii-ME-TRAP) were 2–3-fold higher than those induced with full-length Ii [(fl)human/Ii-ME-TRAP].

The alternative strategy to minimise the potential risk of breaking self-tolerance to human Ii was to design Ii-[antigen] constructs expressing Ii sequences of evolutionarily distant animal species, as recently performed for another molecular adjuvant C4-binding protein^42^. ChAd63 encoding (tr)frog/Ii-ME-TRAP induced particularly high numbers of TRAP-specific CD4^+^ T cells, whereas (tr)trout/Ii-ME-TRAP, (tr)shark/Ii-ME-TRAP and (tr)chicken/Ii-ME-TRAP induced high levels of CD8^+^ T cell responses. Due to very low sequence homology to human/Ii and the ability to enhance CD8^+^ T cells, which are most important in mediating sterile immunity against malaria^43^, shark Ii was further truncated to its TMD (sharkTM/Ii). Sequence analyses showed that sequence homology to humanTM/Ii is low (35% across the 26 amino acids) and, crucially, the largest number of identical consecutive amino acids shared is just three. Therefore, sharkTM fusion to other liver-stage malaria antigens is currently under further investigation and has reached clinical assessment (Silman *et al*. in preparation).

Uncovering the mechanism of action of the Ii as molecular adjuvant has been difficult, given the multiple physiological functions of the invariant chain such as involvement in endosomal sorting, MHC class I and II association, or trimerisation. Holst *et al*. have shown that bone marrow derived DC transduced with adenoviruses expressing antigen fused to Ii have the ability to induce increased antigen-specific CD8^+^ T cell proliferation compared to vectors encoding non-adjuvanted antigen *in vitro*, without any measurable difference in surface expression of MHC class I or costimulatory molecules^44^. CD4^+^ T cell help was not necessary for this enhancement, suggesting that the increased CD8^+^ T cell response is due to a direct effect on transduced DC, leading to more efficient presentation of antigen peptides on MHC class I. In this study, we analysed TRAP expression in transfected A549 cells (human adenocarcinoma epithelial cell line) to determine whether Ii has an impact on intracellular trafficking of the antigen. Indeed, fusion of ME-TRAP to full-length human Ii altered the intercellular localisation of the antigen from being primarily located on the cell surface (ME-TRAP) or being distributed diffusely across the cell (ME-TRAP(w/o_eSP)) to a more perinuclear location ((fl)human/Ii-ME-TRAP). Importantly, truncated and xenogenised Ii retained this characteristic expression pattern, but this effect on intracellular antigen trafficking was reduced when cells were transfected with humanCT/Ii-ME-TRAP, suggesting that the transmembrane domain of the Ii is essential for this effect.

As the majority of transduced cells after an intramuscular injection of viral vectors are most likely muscle cells or fibroblasts, the *in vivo* effect of Ii might not be caused by a direct effect on antigen processing in DC, but rather by a contribution to improved cross-presentation. To allow effective cross-presentation for T cell priming, *in vivo* antigen accumulation and stability has shown to be crucial^32,33^. As oligomers tend to be more stable to proteolysis, we asked whether antigen trimerisation caused by fusion to the Ii could contribute to CD8^+^ T cell enhancement. Although a single point mutation Q64A^34^, reported to disrupt trimerisation, failed to show an effect *in vivo*, subsequent western blot analysis demonstrated Q64A mutation did not affect multimerisation of the Ii/ME-TRAP fusion. The sequence of humanTM/Ii was then systematically mutated to generate variants of humanTM/Ii-ME-TRAP with a reduced tendency to multimerise. The most promising candidate was produced in ChAd63 vectors and found to be much less immunogenic compared to unmutated multimerising humanTM/Ii-ME-TRAP, supporting the notion that the enhancement of CD8^+^ T cell responses by fusion to truncated Ii is in part due antigen stabilisation by multimerisation.

Considering these data, it was hypothesised that CD8^+^ T cell enhancement might be achieved by fusion of the vaccine antigen to any TMD with a strong tendency to multimerise. As the TMD of paramyxoviridae fusion proteins have recently been described to self-associate into trimeric complexes even in the absence of the rest of the protein^35^, one representative sequence (the TMD sequence of the Newcastle disease virus fusion protein, named NC/TMD) was fused to ME-TRAP and tested *in vivo*. Western blot analyses showed that this construct had a strong tendency to multimerise, very similar to the TMD of the Ii (humanTM/Ii). Strikingly, TRAP-specific CD8^+^ T cell responses were as high as those induced by humanTM/Ii-ME-TRAP, suggesting a similarly strong adjuvantation capacity. As this transmembrane domain has been derived from a viral protein, which does not have any closely related eukaryotic proteins (as confirmed by a BLAST analysis), NC/TMD avoids the theoretical risk of autoimmunity associated with Ii derived adjuvants and therefore warrants further investigations. The finding that multimerising TMD seem to be advantageous for antigen-specific CD8^+^ T cell responses when encoded in viral vectors led to the discovery of a whole group of novel T cell adjuvants. Adjuvanticity of the fusion protein TMD of another paramyxoviridae (avian metapneumovirus) as well as the TMD of the influenza A M2 protein, which is known to form a tetramer in the absence of the rest of the M2 protein, confirmed this hypothesis.

The results presented here provide evidence that the ability of Ii fusions to enhance CD8^+^ T cell responses is largely based on its TMD and its outstanding capability to self-associate into trimers, even in the absence of the rest of the Ii protein. Multimerising TMD derived from other transmembrane proteins of non-eukaryotic origin (for example the fusion protein TMD of the Newcastle disease virus), showed a T cell adjuvantation effect, which was as strong as that observed with humanTM/Ii.

Using the viral vector platform in heterologous ChAd63-MVA prime-boost regimens, high frequencies of antigen-specific T cells were observed in clinical trials correlating well with some degree of protective efficacy^1^. Even higher T cell levels may still be required to achieve sterile efficacy against malaria. Whereas most classic adjuvants failed to achieve continued immune enhancement during progression to clinical trial, the full-length Ii as molecular adjuvant has consistently proven its ability to enhance T cell responses with a diverse range of vaccine antigens and in various inbred mouse strains, outbred mice, and also NHP. The results of this study show that (1) the 26 aa long segment of the human Ii (the TMD of its sequence) is sufficient for its adjuvantation effect, (2) xenogenised Ii sequences and (3) other multimerising TMD sequences of non-eukaryotic origin can be used instead of the human sequence. As the induction of strong CD8^+^ T cell responses is crucial for liver-stage immunity, this study strongly supports the progression of adjuvanted ME-TRAP fusion constructs to further trials in NHP and human clinical safety, immunogenicity, and efficacy studies. Furthermore, since cell mediated responses are important for the prevention of many diseases other than malaria, the fusion of Ii to other vaccine antigens may have wide application for other vaccine development programs where potent T cell responses are required.

## Materials and Methods

### Design of Ii-ME-TRAP Fusion Constructs

The ME-TRAP antigen comprises a multi-epitope string (ME) fused to the native *P*. *falciparum* T9/96 strain cDNA sequence encoding TRAP^45^, which is a type 1a membrane protein with a predicted N-terminal signal peptide, a large ectodomain, a transmembrane domain, and a short cytoplasmic C-terminal domain. The multiple epitope string contains a number of HLA restricted immunodominant epitopes from different malaria antigens, BCG or tetanus toxin together with a H-2K^b^ immunodominant epitope from *P*.*berghei* CSP which was initially included to enable immunological assessment in BALB/c mice^45^. The Ii (modified as indicated in the text and graphs) was fused to the N-terminal end of ME-TRAP. When fused to Ii sequences, the nucleotides 1–75 of TRAP were deleted from the construct, which encodes a predicted signal peptide in order to prevent hydrolysis of the Ii from TRAP (if signal peptide cleavage were to occur). This deletion within ME-TRAP itself did not have an impact on immunogenicity when expressed in ChAd63 vectors as shown in Figure S1. A mixture of gene synthesis and conventional cloning was used to make in frame fusions of these constructs.

### Construction of Recombinant Adenovirus Vectors

The above mentioned [Ii]-ME-TRAP constructs were subcloned into a transgene expression cassette comprising a modified human cytomegalovirus major immediate early promoter (CMV promoter) with tetracycline operator (TetO) sites^46^. The cassettes were inserted into the E1 locus of an E1/E3-deleted and E4-modified genomic clone of ChAd63^47^ using site-specific recombination^46^ with the viruses rescued and propagated in T-REx-293 cells, purified by CsCl gradient ultracentrifugation and titred as previously described^48^. Doses for vaccination were based on infectious units (IU) and not viral particles as it is infectivity, rather than viral particle number, which is correlated with immunogenicity^48^. ChAd63 particle-to-infectious unit (P:I) ratios were in the range 50–120.

### Construction of Recombinant MVA

[Ii]-ME-TRAP constructs were sub-cloned into an orthopoxviral shuttle plasmid under control of the vaccinia virus p7.5 promoter. The cassette was introduced into the thymidine kinase (TK) locus of MVA by recombination in transfected and infected chick embryo fibroblast (CEF) cells followed by transient selection with a GFP marker gene. The resulting markerless viral recombinants were plaque-purified, amplified in CEF, and titrated using an immunostaining plaque assay according to standard methods. The identity and purity of the isolates were verified by PCR. Doses for vaccination were based on plaque forming units (PFU).

### Ethics Statement

All animal work was conducted in accordance with the UK Animals (Scientific Procedures) Act 1986 and approved by the University of Oxford Animal Care and Ethical Review Committee for use under Project License 30/2889 and P9804B4F1. Animals were group housed in individually ventilated cages under specific pathogen free conditions, with constant temperature, humidity and with a 12:12 light-dark cycle (8 am to 8 pm). For induction of short-term anaesthesia, animals were either injected intramuscularly (i.m.) with xylazine and ketamine or anaesthetized using vaporized IsoFlo®. All animals were humanely sacrificed at the end of each experiment by an approved Schedule 1 method. All efforts were made to minimize suffering.

Ethical approval for use of male rhesus macaques was granted by the University of Wisconsin-Madison IACUC (termed Animal Care and Use Committee) and granted protocol number G00713-0-05-13. The Wisconsin National Primate Research Center (WNPRC) executed the study to honour the fee-for service agreement between the University of Wisconsin, USA and the University of Oxford, UK. Processing of blood samples, fresh enzyme linked-immunospot (ELISpot) and fresh intracellular cytokine staining (ICS) were performed at the University of Wisconsin, with serum and frozen PBMC shipped to Oxford for additional immunological investigations. Macaques were housed in standard stainless steel primate cages (Surburban Surgical, Chicago, IL): They were fed twice daily with commercial chow (20% protein primate diet, catalog no. 2050; Harlan Teklad, Madison, WI) and also given a variety of fruit enrichment in the afternoons. Housing rooms were maintained at 18 °C to 24 °C (65° to 75 °F), 30 to 70% humidity, and on a 12:12 light-dark cycle (on, 6:00 a.m.; off, 6:00 p.m.). As a source of environmental enrichment puzzle feeders of various sorts were provided at least twice per week, destructible foraging one time per week in addition to daily fruit or yoghurt cup. All procedures were performed by highly trained animal technicians or veterinary staff. To minimize distress and suffering during vaccination or blood withdrawal, animals were anesthetised for all procedures with blood draws limited to the maximum volume allowed over the duration of the experiment. All animals were returned into the colony at the end of the study for use in other experiments or breeding programs (as appropriate).

### Animals and Immunisations

Female C57BL/6JOlaHsd (C57BL/6) or BALB/cOlaHsd (BALB/c) mice aged at least 6 weeks (Envigo, UK), were given intramuscular (IM) immunisations into the musculus tibialis with a total volume of 50 µL of vaccine, diluted in endotoxin-free PBS using a 29 G 0.5 mL insulin syringe (BD). Each figure is an independent *in vivo* experiment. Because of the variability between *in vivo* experiments due vaccination preps and ICS performed on different days, replicate experiments have not been pooled together, instead the data is representative of a minimum of at least 2 experiments comparing the effect of the adjuvant against the unadjuvanted ME-TRAP control.

Rhesus macaques were screened for background ELISpot responses to TRAP and ME peptides (no response in any animal was detected) and divided into two experimental groups, based on achieving approximate equivalence of age and weight. The control (ME-TRAP) group comprised 6 animals with a median weight of 7.76 kg (range, 4.45–12.22) and median age of 4.3 years (range, 3.1–5.7); and the test (hIi-ME-TRAP) group comprised 6 animals with a median weight of 6.45 kg (range, 4.36–8.82) and a median age of 3.8 years (range 3.1–5.2). Animals received i.m. immunisations with 5 × 10^7^ iu ChAd63.ME-TRAP (corresponding to 2.9 × 10^9^ vp) or 5 × 10^7^ iu ChAd63.hIi-ME-TRAP (corresponding to 3.7 × 10^9^ vp) in a total of 0.3 ml of endotoxin-free PBS into the left deltoid muscle. At week 8 after priming, animals that had received ChAd63.ME-TRAP were boosted with 8 × 10^7^ plaque forming units (PFU) MVA.ME-TRAP and animals that had received ChAd63.hIi-ME-TRAP were boosted with 8 × 10^7^ PFU MVA.hIi-ME-TRAP. These boost vaccinations were given into the right (contralateral) deltoid muscle. Blood samples were taken pre-vaccination, on the day of vaccination (week 0) and at 2, 4, 6, 8, 9, 11 and 16 weeks after the first vaccination.

### Antigens for *In Vitro* Restimulation

Peptides used in immunological assays were purchased from commercial suppliers (NeoScientific, Woburn, Massachusetts, USA; Mimotopes, Wirral, UK; or Thermo Fisher Scientific). Peptides overlapping by ten amino acids (aa) for the entire protein sequence of *P*. *falciparum* TRAP (greater than 80% purity) or the human Ii (crude) were used. In some experiments using BALB/c mice, cells were stimulated with the immunodominant H-2^d^ restricted epitope of ME-TRAP (Pb9) (SYIPSAEKI). Peptides were reconstituted in DMSO at 50–100 mg/mL depending on their solubility and combined into a final peptide pool for cellular assays with DMSO at a final concentration of less than 1%. For the human Ii, several sub-pools were prepared: hIi-Cytoplasmic-Tail (Ii-1 to Ii-11), hIi-TMD (Ii-12 to Ii-16), and hIi-Luminal-Tail (Ii-17 to Ii-56).

### Intracellular Cytokine Staining (ICS)

PBMC or splenocytes were plated in 96-well round bottom plates and stimulated by the addition of individual Pb9 peptide or pools of overlapping 20mers covering the whole protein (TRAP or Ii as indicated) at a final concentration of 2 µg/mL in the presence of 1 µg/mL BD GolgiPlug™, and incubated for 6 hours (37 °C, 5% CO_2_). After cell surface labelling with anti-CD4-e450 and anti-CD8-PerCP/Cy5.5 antibodies (Affymetrix eBioscience) as well as LIVE/DEAD® Fixable Aqua Dead Cell Stain Kit (Thermo Fisher Scientific), cells were fixed with neutral buffered formalin solution containing 4% formaldehyde (Sigma Aldrich) for 5 minutes at 4 °C. Then, intracellular staining was performed with anti-TNF-Alexa488, anti-IL-2-PE and anti-IFN-γ-Alexa647 antibodies (Affymetrix eBioscience) diluted in BD Perm/Wash buffer. Flow cytometry data was analysed using a BD LSR II Flow Cytometer with BD FACSDIVA (Becton Dickinson) and FlowJo (Tree Star) software. Antigen-specific cells were identified by gating based on size, doublet negative, live cells and either CD4^+^ or CD8^+^ surface expression. Background responses in unstimulated wells were subtracted from responses of stimulated T cells before statistical analysis in Prism 6.07 (GraphPad).

### ELISpot

Macaque IFN-γ ELISpot assays were performed as previously described^49^ using precoated ELISpot^PLUS^ kits according to the manufacturer´s recommendation (Mabtech, USA). All tests were performed in duplicate using a TRAP peptide pool at final concentration of 5 µg/mL with incubation for 12–18 hours at 37 °C in 5% CO_2_. Enumeration and calculations were performed as above. A response was considered positive if the spot forming units (SFU) exceeded mean background plus two standard deviations (SD) and was >50 SFU per 10^6^ cells.

### Antibody Responses

Antibody responses to TRAP were measured using a luciferase immunoprecipitation system (LIPS) as previously described^50^. The assay is based on binding of immobilised antibodies to a fusion protein of TRAP (*P*. *falciparum* 3D7 sequence) (human Ii or macaque Ii) and Renilla luciferase (rLuc). Briefly, serum samples were incubated for 1 hour with a cell lysate from 293 cells transfected with a TRAP-rLuc (hCD74-rLuc or macCD74-rLuc) expression plasmid, prior to incubation with Protein A/G UltraLink Resin beads (Thermo-Scientific) in MultiScreen HTS membrane Barex plates (Millipore) for 1 hour. Unbound lysate and antibodies were removed by washing the plates prior to quantification of bound rLuc activity using Renilla luciferase assay system (Promega) and a Varioskan Flash luminometer (Thermo). Antibody levels are expressed as log10 luminescence units.

### Western Blot

HEK293A cells were seeded in 6-well plates (400,000 cells/well in 2 mL) and transfected with 3 µg plasmid DNA using Lipofectamine 2000. The following day, cells were lysed in 250 µL cell lysis buffer (incl. proteinase inhibitor, cOmplete, Mini, EDTA-free, Roche) for 20 min on ice and centrifuged at 16,000 g for 10 min. The supernatant was stored at −20 °C for up to 10 days. For western Blot analyses, lysates were thawed on ice, mixed with 4 × LDS loading buffer ± 10% 2-Mercaptoethanol (for reducing conditions, Sigma) and incubated at 85 °C for 5 min. Samples and protein ladder (HiMark™ Pre-Stained HMW Protein Standard, Thermo Fisher Scientific) were then run on NuPAGE™ Novex™ 4–12% Bis-Tris Midi Protein Gels (Thermo Fisher Scientific) according to the manufacturer’s instructions.

Proteins were blotted using Trans-Blot® Turbo™ Midi Nitrocellulose Transfer Packs (Bio-Rad) and the Trans-Blot® Turbo™ Transfer System (Bio-Rad) according to the manufacturer’s instructions. Subsequently, the membrane was incubated in 3% BSA/PBS for 1 h at RT, followed by incubation with primary antibodies diluted in 3% BSA for 1 h at RT. Membranes were then incubated with mouse polyclonal serum against ME-TRAP (1:1,000 in 3% BSA), washed twice with PBS-Tween (10 min each), and incubated with Peroxidase AffiniPure Donkey Anti-Mouse IgG (H + L) (Jackson ImmunoResearch Laboratories Inc., West Grove, PA, USA) diluted 1:10,000 in 3% BSA. Membranes were washed with PBS-Tween and water before developing with Thermo Scientific™ SuperSignal™ West Pico Chemiluminescent Substrate (Thermo Fisher Scientific) and imaging using a ChemiDoc™ MP System (Bio-Rad) according to the manufacturers’ instructions.

## Electronic supplementary material

Supplementary data
