# Supplementary material for: Development of a Molecular Adjuvant to Enhance Antigen-Specific CD8+ T Cell Responses
Source: Sci Rep. 2018 Oct 9;8:15020. doi: 10.1038/s41598-018-33375-1 (PMC6177389; doi:10.1038/s41598-018-33375-1)
Supplement: Supplementary file 1 — Supplementary data [file 41598_2018_33375_MOESM1_ESM.pdf]

Development of a Molecular Adjuvant to Enhance Antigen-Specific CD8$^+$ T Cell Responses

Benedict R. Halbroth*, Sarah Sebastian, Hazel Poyntz, Migena Bregu, Matthew G. Cottingham, Adrian V. S. Hill, Alexandra J. Spencer*

The Jenner Institute, University of Oxford, ORCRB, Roosevelt Drive, Oxford, United Kingdom

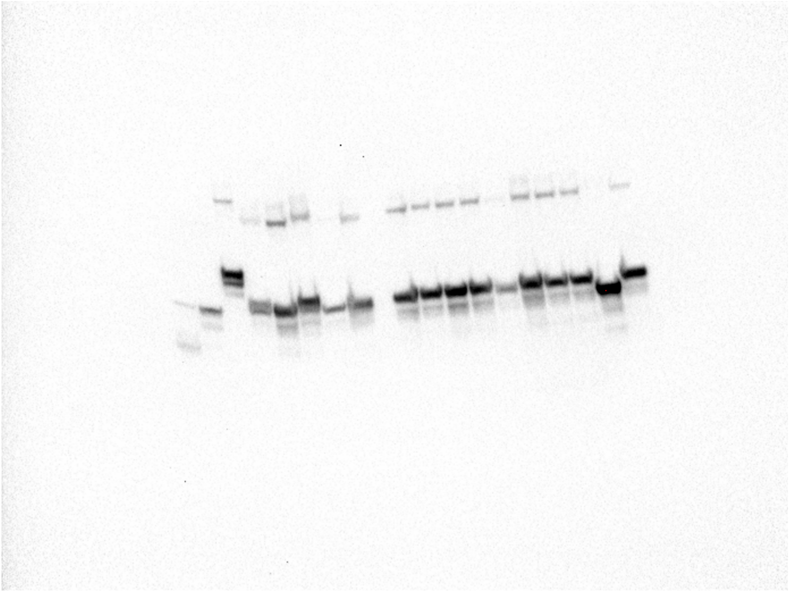

Figure 6

Full western blot image from Figure 6.

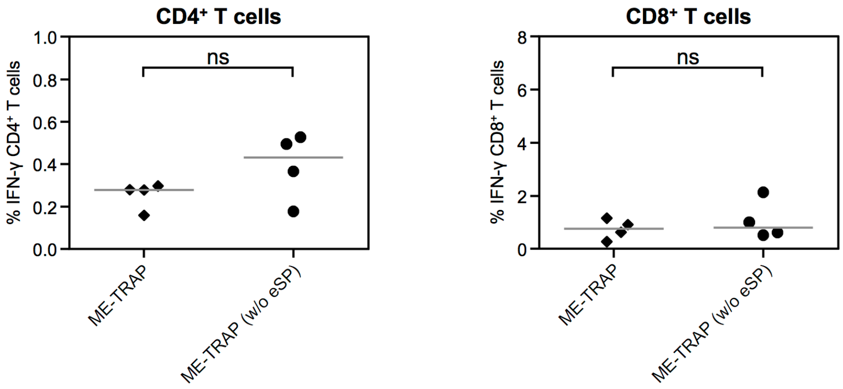

Figure S1

Effect of endogenous signal peptide (eSP) removal from TRAP. C57BL/6 mice were immunised with 10^8 IU ChAd63 vectors encoding ME-TRAP with or without its endogenous signal peptide. Spleens were harvested two weeks later. $CD4^+$ and $CD8^+$ T cell responses to a TRAP peptide pool are shown by measuring intracellular IFN-γ using flow cytometry. Single points represent T cell responses of individual mice and lines indicate the median response per group. Data was analysed with a one-way analysis of variance. No statistical difference was found between the groups.

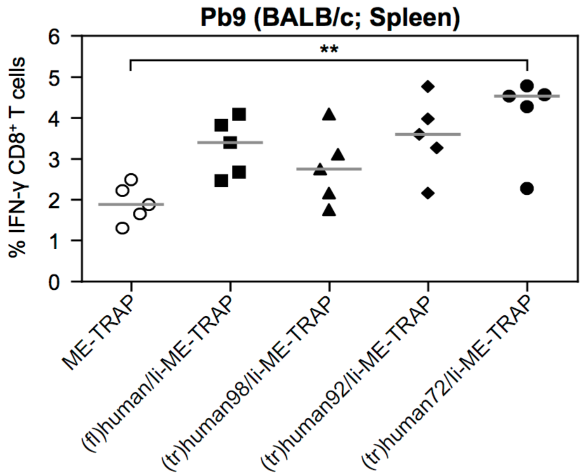

Figure S2

Immune response against Pb9. In the same experiment as described in Figure 1B, $CD8^+$ T cell responses to Pb9 measured by intracellular staining for IFN-γ by flow cytometry is shown. Single points represent T cell responses of individual mice and lines indicate the median response per group. Data was analysed with a one-way analysis of variance with Dunn's multiple comparison post-test. Asterisks denote the level of statistical significance when compared to the control ME-TRAP vaccinated group (**, p<0.01).

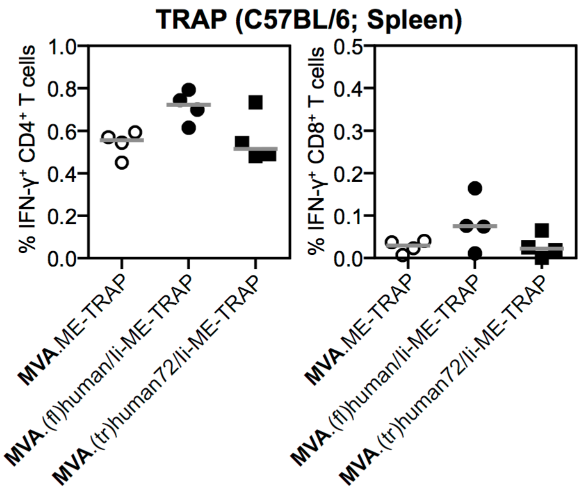

Figure S3

MVA induced immune responses. Four C57BL/6 mice per groups were immunised with 10^6 PFU MVA unfused or adjuvanted ME-TRAP as indicated on the x-axis. Spleens were harvested one week later. $CD4^+$ and $CD8^+$ T cell responses to a TRAP peptide pool are shown by measuring intracellular IFN-γ using flow cytometry. Single points represent T cell responses of individual mice and lines indicate the median response per group. Data was analysed with a one-way analysis of variance with Dunn's multiple comparison post-test. No statistical difference was found between the groups.

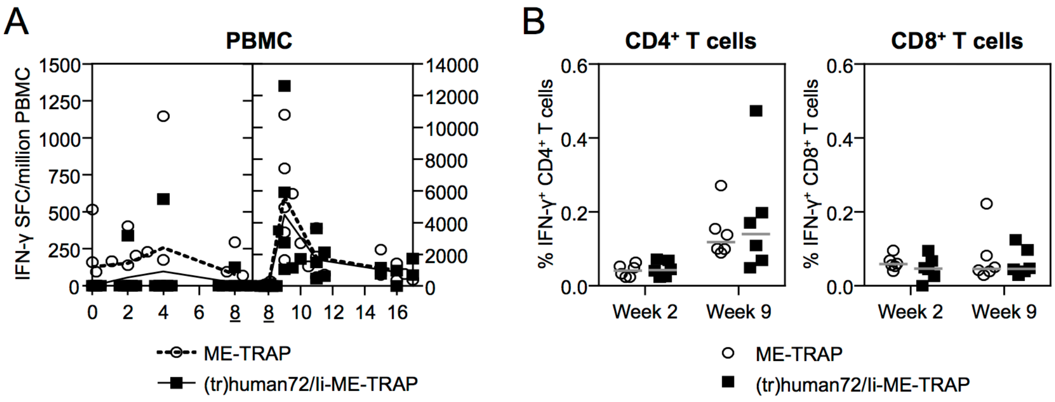

Figure S4

TRAP-specific response in non-human primates. Male rhesus macaques were vaccinated IM with either ChAd63 encoding ME-TRAP followed eight weeks later by MVA.ME-TRAP, or ChAd63.(tr)human72/Ii-ME-TRAP followed eight weeks later by MVA.(tr)human72/Ii-ME-TRAP. Blood samples were taken on the day of vaccination, weeks, 2, 4, 8 following ChAd63 vaccination on d 1 (9), 3 (11) and 8 (16) weeks post MVA vaccination. (A) Graphs represent the TRAP-specific response in PBMC measured by IFN-γ ELISpot assays over the course of the experiment. Single points represent T cell responses of individual macaques and connecting lines indicate the group´s mean response. (B) Graphs represent the TRAP-specific $CD4^+$ and $CD8^+$ T cell response at week 2 and week 9 measured by ICS. Single points represent T cell responses of individual animals and lines indicate the median response per group. Data was analysed with a one-way analysis of variance. No statistical difference was found between the groups.

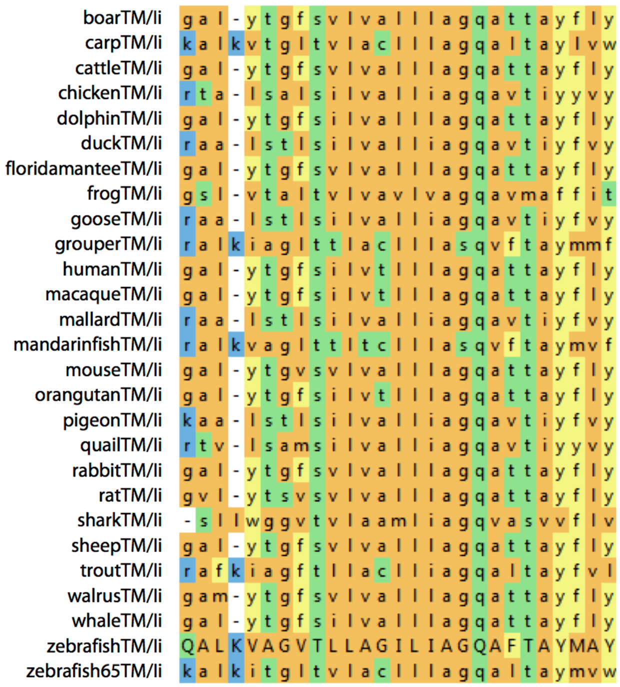

Figure S5

MUSCLE alignment of Ii transmembrane domains.

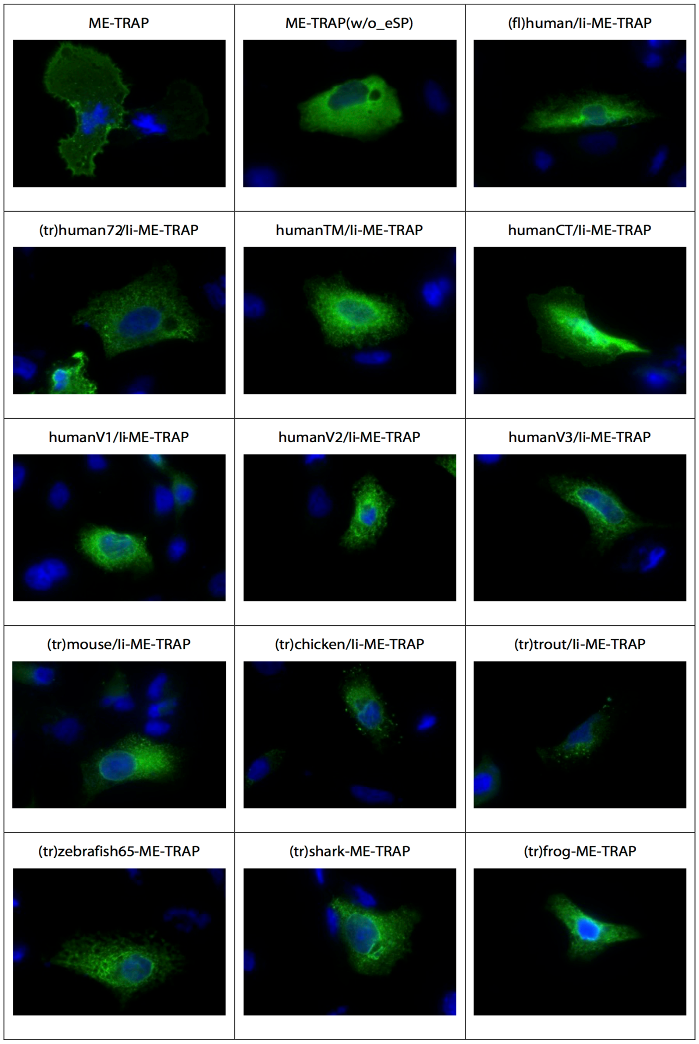

Figure S6

Invariant chain fusion alters the pattern of TRAP expression. A549 cells were transfected with pENTR4-LPTOS plasmids encoding different Ii-ME-TRAP fusion constructs, incubated overnight prior to staining for TRAP expression (green). The nucleus was stained with DAPI (blue). Immunofluorescence images in 100× magnification are shown.

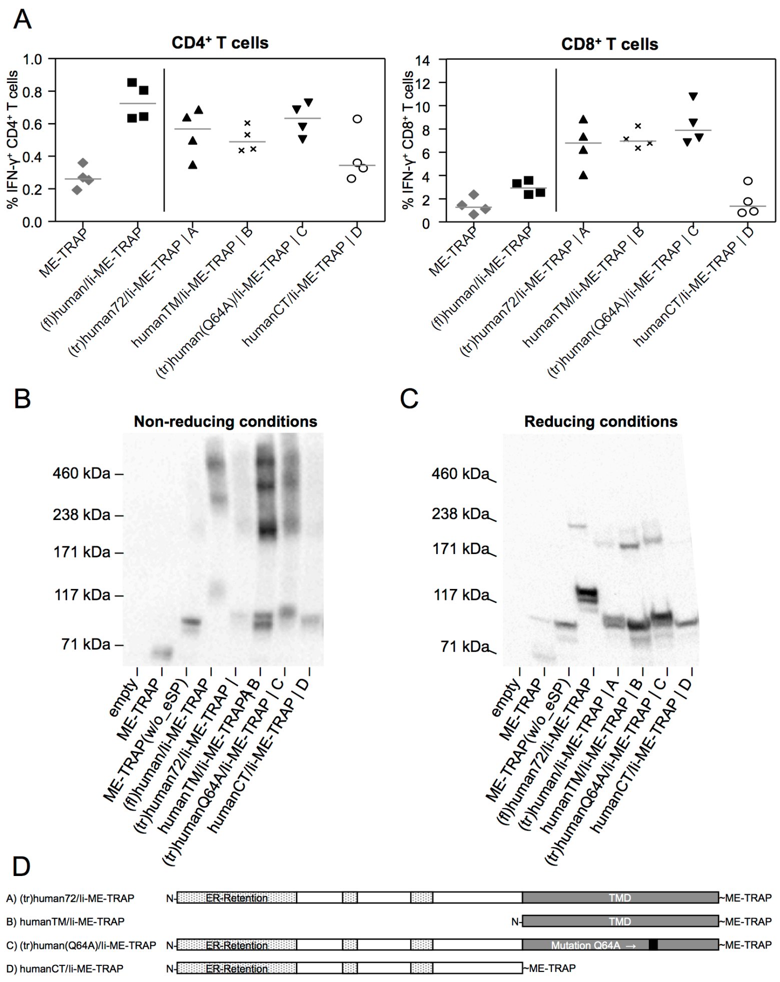

Figure S7

Analyses of (tr)humanQ64A/Ii fused to ME-TRAP. (A) C57BL/6 mice were immunised with 10^7 IU ChAd63 vectors and spleens were harvested two weeks later. T cell responses to a TRAP peptide pool were analysed by ICS. The percentage of $CD4^+$ and $CD8^+$ T cells positive for IFN-γ are shown. Single points indicate T cell responses of individual mice and lines denote the median response per group. (B and C) Western blot of unfused and adjuvanted ME-TRAP constructs. HEK293 cells were transfected with plasmid DNA and lysed one day later. Lysates were then analysed using polyacrylamide gels and western blots stained with polyclonal serum against ME-TRAP (B) Western blot of lysates in non-reducing conditions: LDS loading buffer, 85°C, 5 min. (C) Western blot in reducing conditions: LDS loading buffer + 10% 2-Mercaptoethanol, 85°C, 5 min. (D) Illustration of different Ii versions compared in this experiment.

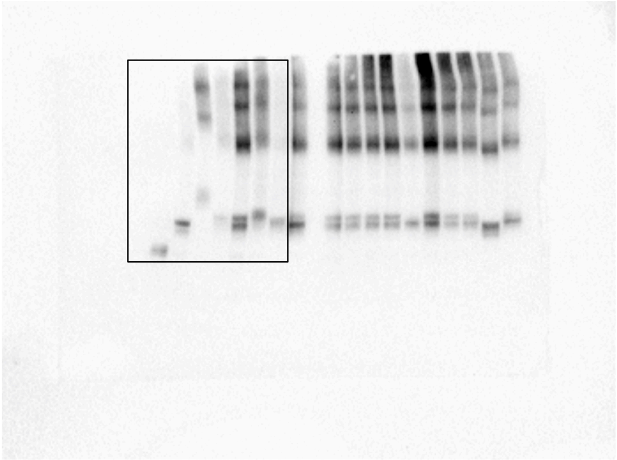

Figure S7b

Full western blot of images of Figure S7b

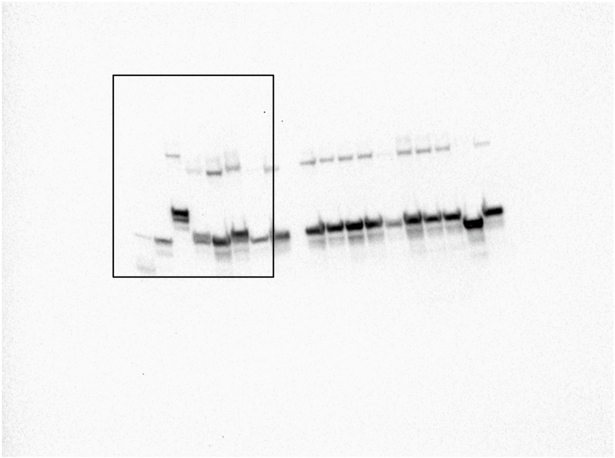

Figure S7c

Full western blot of images of Figure S7c

Name	GenBank Identifier/ NCBI Reference Sequence	Homology with (fl)human/Ii	Homology with (fl)mouse/Ii
**(fl)human/Ii**	**NP_004346**	**100%**	**73.8%**
(fl)orangutan/Ii	NP_001124630.1	98.7%	32.2%
(fl)macaque/Ii	XP_002804624.1	86.8%	39.1%
(fl)floridamantee/Ii	XP_004384950.1	78.3%	29.1%
(fl)cat/Ii	XP_003981534.1	77.3%	33.6%
(fl)walrus/Ii	XP_004402873.1	79.5%	29.6%
**(fl)mouse/Ii**	**NP_034675**	**73.8%**	**100%**
(fl)rat/Ii	NP_037201.1	74.9%	86%
(fl)whale/Ii	XP_004280397.1	76.2%	75.9%
(fl)dolphin/Ii	XP_004326859.1	77.1%	75.5%
(fl)boar/Ii	NP_998939.1	77.6%	77.4%
(fl)sheep/Ii	XP_004009025.1	78.4%	78.7%
(fl)cattle/Ii	BAA12156.1	79.9%	79.2%
(fl)rabbit/Ii	XP_002710211.1	73.6%	76.2%
(fl)duck/Ii	AEB71788.1	45.5%	42.9%
(fl)mallard/Ii	XP_005010602.1	42.9%	40.2%
(fl)goose/Ii	ADK11994.1	44.6%	43.3%
(fl)quail/Ii	ADG01642.1	41.1%	38.8%
(fl)chicken/Ii	NP_001001613.1	40%	37.2%
(fl)pigeon/Ii	AAX47311.1	46.5%	44.9%
(fl)frog/Ii	NP_001184041.1	27.8%	28.5%
(fl)grouper/Ii	AEA39679.1	23.7%	22.6%
(fl)mandarinfish/Ii	AAS77256.1	22.5%	22.5%
(fl)zebrafish65/Ii	NP_571665.1	20.1%	20.7%
(fl)carp/Ii	AEZ67439.1	19.1%	18.6%
(fl)zebrafish/Ii	NP_571447.1	16.4%	16.9%
**(fl)trout/Ii**	**NP_001117913.1**	**20%**	**18.4%**
**(fl)shark/Ii**	**AEX34752.1**	**21.9%**	**22.1%**

Table S1

Homology analysis of full-length Ii sequences. Full-length Ii sequences of various species were compared for homology to the full-length human and mouse Ii sequences. Entitled name, GenBank identifier/NCBI Reference Sequence, and sequence homology with (fl)human/Ii or (fl)mouse/Ii sequence are listed. Homology was analysed using the ClustalW algorithm in DNAStar® Megalign. Highlighted constructs were fused to the N-terminal end of ME-TRAP and also produced in ChAd63 vectors.

Name	GenBank Identifier/ NCBI Reference Sequence	Homology with (tr)human72/Ii	Homology with (tr)mouse/Ii
**(tr)human72/Ii**	**NP_004346**	**100%**	**83.6%**
(tr)orangutan/Ii	NP_001124630	98.6%	81.8%
(tr)boar/Ii	NP_998939	91.1%	87.3%
(tr)macaque/Ii	XP_002804624	90.3%	81.8%
(tr)cattle/Ii	BAA12156	89.3%	85.5%
(tr)dolphin/Ii	XP_004326859	89.3%	81.8%
(tr)cat/Ii	XP_003981534	87.5%	89.1%
(tr)rabbit/Ii	XP_002710211	87.5%	87.3%
(tr)sheep/Ii	XP_004009025	87.5%	87.3%
(tr)walrus/Ii	XP_004402873	87.5%	85.5%
(tr)whale/Ii	XP_004280397	87.5%	81.8%
(tr)floridamantee/Ii	XP_004384950	85.7%	89.1%
**(tr)mouse/Ii**	**NP_034675**	**83.6%**	**100%**
(tr)rat/Ii	NP_037201	78.6%	87.3%
(tr)pigeon/Ii	AAX47311	50.0%	52.0%
(tr)duck/Ii	AEB71788	49.1%	55.8%
(tr)quail/Ii	ADG01642	43.6%	40.0%
**(tr)chicken/Ii**	**NP_001001613**	**42.9%**	**40.0%**
(tr)goose/Ii	ADK11994	41.8%	53.8%
**(tr)zebrafish/Ii**	**NP_571447**	**37.0%**	**37.3%**
(tr)mallard/Ii	XP_005010602	32.8%	40.0%
(tr)zebrafish65/Ii	NP_571665	32.8%	38.9%
**(tr)trout/Ii**	**NP_001117913**	**31.6%**	**34.5%**
(tr)carp/Ii	AEZ67439	31.6%	50.0%
(tr)grouper/Ii	AEA39679	30.0%	33.3%
(tr)mandarinfish/Ii	AAS77256	27.9%	29.6%
**(tr)frog/Ii**	**NP_001184041**	**27.6%**	**37.0%**
**(tr)shark/Ii**	**AEX34752**	**24.6%**	**36.4%**

Table S2

Homology analysis of truncated Ii sequences. Ii sequences from a variety of animal species were truncated corresponding to the length of (tr)human72/Ii and compared for homology to truncated human and mouse Ii sequences. Entitled name, GenBank identifier/NCBI Reference Sequence, and sequence homology with (fl)human/Ii or (fl)mouse/Ii sequence are listed. Homology was analysed using the ClustalW algorithm in DNAStar® Megalign. Highlighted constructs were fused to the N-terminal end of ME-TRAP and produced in ChAd63 vectors.
